# Transition state characteristics during cell differentiation

**DOI:** 10.1371/journal.pcbi.1006405

**Published:** 2018-09-20

**Authors:** Rowan D. Brackston, Eszter Lakatos, Michael P. H. Stumpf

**Affiliations:** 1 Centre for Integrative Systems Biology and Bioinformatics, Department of Life Sciences, Imperial College London, London, United Kingdom; 2 School of BioScience and School of Mathematics and Statistics, University of Melbourne, Melbourne, Australia; Oxford, UNITED KINGDOM

## Abstract

Models describing the process of stem-cell differentiation are plentiful, and may offer insights into the underlying mechanisms and experimentally observed behaviour. Waddington’s epigenetic landscape has been providing a conceptual framework for differentiation processes since its inception. It also allows, however, for detailed mathematical and quantitative analyses, as the landscape can, at least in principle, be related to mathematical models of dynamical systems. Here we focus on a set of dynamical systems features that are intimately linked to cell differentiation, by considering exemplar dynamical models that capture important aspects of stem cell differentiation dynamics. These models allow us to map the paths that cells take through gene expression space as they move from one fate to another, e.g. from a stem-cell to a more specialized cell type. Our analysis highlights the role of the transition state (TS) that separates distinct cell fates, and how the nature of the TS changes as the underlying landscape changes—change that can be induced by e.g. cellular signaling. We demonstrate that models for stem cell differentiation may be interpreted in terms of either a static or transitory landscape. For the static case the TS represents a particular transcriptional profile that all cells approach during differentiation. Alternatively, the TS may refer to the commonly observed period of heterogeneity as cells undergo stochastic transitions.

## Author summary

Current emphasis on single cell analysis, especially in the context of the human and mouse cell atlas projects, is on characterizing the transcriptomic signatures of different cell states. This is clearly of great importance, as even the number of different cell types, e.g. in humans, is not known with any satisfying degree of certainty. There are enormous challenges in mapping these states, but this will still only provide a partial answer. Importantly, the way in which cells differentiate, and the way in which gene expression changes over the course of differentiation will still be unknown. Here we use a dynamical systems perspective to consider the nature of, and dynamics during, the transition between different cell types (or cell states). We show how the developmental landscape (in Waddington’s sense) and the nature of the transition states change in response to external stimuli and discuss this in the context of stem cell differentiation (as well as its potential reversal). In particular, we discuss how the nature of the landscape at the transition state, as well as the presence of non-gradient dynamics, has strong implications for the identifiability of differentiation dynamics from experimental data.

## Introduction

Cells are not inert objects. They have finite lifetimes with typically well defined origins and ends. And over the course of their lifetime—which lasts anything from minutes to many years—change in response to environmental, physiological and, potentially, developmental signals [1]. Some of these changes are minor, e.g. changing the expression of certain proteins in response to an environmental signal, or the activity of an enzyme as part of metabolism. Others relate to longer-term, less reversible, or more profound changes in cell state; including commitment to replication, apoptosis, or differentiation. The former set of changes can be viewed as *tactical* decisions which are made in response to (typically transient) changes in a cell’s environment [2], whereas the latter are of more *strategic* importance for the cell and, where relevant, potentially the organisms as a whole [3, 4].

In humans, a single fertilized egg cell eventually gives rise to some 35 trillion cells in the adult. How many cell types there are remains an unanswered question, but some aspects of the process by which an omni-potent stem cell differentiates into a more specialized cell are now becoming clearer. Remodeling of the gene regulatory networks—typically in response to signaling events—change the transcriptional program of the cell, thereby leading to a concomitant change in cell phenotype/state. We will here assume for ease of argumentation, that the molecular state of a cell can reflect the true state of the cell, its *phenotype*, but stress that this may only be a poor substitute for a more direct biological or phenotypic characterization.

The popular metaphor of the *Waddington’s epigenetic landscape* has come to predominate much of the discussion about cell differentiation processes: cells are described as marbles rolling through a landscape of hills and valleys drawn towards local points of minimum elevation [1]. An individual ball starts its journey in a valley at the back of the landscape and as it progresses forward (the passage of time in the original formalism) and downward; it might face branching points along the path, representing the series of (typically binary) fate choices made by a developing cell. Every point a ball travels through represents a cellular state, for example a specific level of expressed RNA or protein. Although the number of possible states is theoretically infinite, the number of phenotypes observed in actual cells are often very limited. In this view, the final basins of low elevation where a high proportion of cells end up correspond to these experimentally observable, terminal cell types.

The key insight offered by the landscape is to illuminate how genetically identical cells can attain distinct phenotypes following differentiation, and furthermore how these phenotypes persist in daughter cells. While such persistence and memory effects are understood to result from the epigenetic and proteomic state of the cells, the landscape itself is widely regarded to be shaped by the underlying gene regulatory network [5, 6]. In this way, models describing the co-regulation and interactions between different genes may also be understood to describe the epigenetic mechanisms underlying persistent stem cell differentiation.

The landscape notation has been widely used as a qualitative way of understanding and illustrating the dynamics driving development [7–9]. Moreover, even though Waddington may not have intended his landscapes to be any more than a conceptual tool, landscapes can be given quantitative meaning: exploring the behaviour of the underlying network in terms of a probabilistic landscape framework. A succession of studies have recently proposed different approximations to potential functions that can serve as mathematical descriptions of the epigenetic landscape for a cell fate regulatory network. Here the elevation of the surface reflects the probability of observing a particular state in phase space [6, 10–14]: states that have the highest probability locally will have lower potential and hence will act as the valley-bottoms on the landscape, surrounded by a basin of attraction, which in this picture would correspond to cells with slightly different states but exhibiting the same phenotype.

Even though the landscape depiction might be adopted to many decision making processes over the lifetime of a cell, its major uses are still in describing development and stem cell differentiation processes [15, 16]. In this context the final attractors of the landscape are differentiated cell types with well-defined patterns of robust gene expression, and differentiation occurs through transitions along the surface, while some cells might reside or return to the original basin of the pluripotent phenotype.

Mathematically, we can draw on a vast body of work to characterize the cell states defined in terms of steady-states of molecular concentrations. For the sake of clarity, we consider *X* to denote the state of the system (e.g. the whole set of mRNA and/or protein abundances), which evolves according to the stochastic differential equation (SDE)

$$\begin{array}{c} \text{d}X=f\left(X;\theta \right)\;\text{d}t+g\left(X;\eta \right)\;\text{d}W_{t}, \end{array}$$ (1)

where *f*(*X*; *θ*) describes the deterministic dynamics of the system, and *g*(*X*; *η*) d*W*_*t*_ captures the stochastic components of the dynamics; these functions being parametrized by (typically vector-valued) *θ* and *η*, respectively. If the latter can be ignored we recover a more conventional ordinary differential equation (ODE), commonly written as

$$\begin{array}{c} \frac{\text{d}X}{\text{d}t}=f\left(X;\theta \right). \end{array}$$ (2)

This, setting the left-hand side equal to zero, allows us to solve for the (deterministically) stationary states of the system, which if they are stable, i.e. if they are *attractors*, we assume correspond to distinct cell states.

While many stationary states of the system may describe the recognizable, robust cell phenotypes, others may correspond to the so-called intermediate cell states (ICS) [17]. These are states with a particular molecular phenotype, but without the particular function that usually accompanies traditionally defined cell types. ICS have been observed experimentally in epithelial-mesenchymal transition [18] and hematopoietic differentiation [19, 20]. Here, by examining models for stem cell differentiation from a dynamical systems perspective, we explore how the related concept of *transition states* (TS) may arise, and how their properties are affected by external signals.

If the set {*X*_1_, …, *X*_*q*_} denotes the set of stable stationary/attractor states of (2), we identify them with the valleys/local minima in the corresponding epigenetic landscape for the system, which in turn correspond to robustly defined cellular phenotypes. Classifying the stability of these solutions (e.g. in the presence of stochastic effects), and the basins of attraction has been one of the long-standing problems in dynamical systems theory. Closely linked to it is the question of how different stationary points can be reached from one another: is there a particularly favored path that the system traverses as it moves from, say *X*_*i*_ to *X*_*j*_ (1 ≤ *i*, *j* ≤ *q*)? In general, any two stable stationary states of a dynamical system must be separated by an unstable stationary state. As will be discussed, such transition states may sometimes correspond to the experimentally observed ICS.

In the context of stochastic dynamical systems, the most probable paths between stationary states correspond to those that minimize the *action* associated with them [21]: the so-called *minimum action paths* (MAP). This concept has allowed several previous investigations to shed light on the typical routes taken through gene expression space between phenotypes in stochastic models of cellular differentiation. For example [22] obtained the most probable paths between phenotypes in a 52 gene network describing stem cell behaviour. These paths were obtained via the method detailed in [23], wherein a kinetic path framework is developed both to obtain most likely paths and to calculate a landscape. Similarly, in [14], the geometric minimum action method [24] is used to obtain the most probable transition paths in a stem cell differentiation model, while [25] obtained paths for a model of epithelial-mesenchymal transition. In this work we use a similar approach, obtaining the most probable paths in the forward (differentiation) and reverse (reprogramming) directions, but with a particular emphasis on the transition states through which these paths pass.

Below we will use a set of illustrative examples that allow us to study the transitions between stationary solutions of (stochastic) differential equations, with an emphasis on dynamical systems in the context of stem cell differentiation. In particular we shall be investigating the concept of *transition states* (TS), as recently discussed and reviewed by [5]. For representative model systems we discuss two competing definitions for the transition state in the context of static and transitory landscapes. For static landscapes we shall employ the concept of the MAP to examine typical transition paths. We first discuss the properties of transition states for an exemplar “toy” system before applying the same analysis to a developmental model. In particular we will aim to answer four linked questions:

- What defines a TS for a stochastic dynamical system?
- How long does the system spend at the TS?
- Is the TS the same for both differentiation and reprogramming?
- How does the epigenetic landscape, and especially the TS change, in response to external signals?

## Results

### The potential landscape and transition states in a bistable system

In order to exemplify some key properties of stochastic dynamical systems in the context of potential landscapes we provide a simple model that exhibits some of the hallmarks found in real developmental systems. The model we provide is, however, not intended to represent any particular biological process, but serves as a simple example in which potential landscapes and transition states may be described. We will use this model to firstly demonstrate the distinction between gradient and non-gradient dynamics, before examining two definitions for the concept of the transition state.

#### Limitations to gradient based dynamics

The SDE defined in (1) above describes a dynamical system with both deterministic and stochastic components. In general, the deterministic forcing vector *f*(*X*; *θ*) of such SDEs may be decomposed into the sum of the gradient of a potential, ∇*U*, and a remaining component often referred to as the *curl* or *flux* [26, 27]. This decomposition may be written as,

$$\begin{array}{c} f\left(X;\theta \right)=-\nabla U\left(X;\theta \right)+f_{U}\left(X;\theta \right). \end{array}$$ (3)

The underlying concept is that the potential describes the portion of the dynamics that determines the basins of attraction (and thus the most probable states). This potential thereby gives an intuitive explanation for the observed behaviour; the dynamical system will tend to stay near to the minima in the potential and will move away from regions of higher potential.

A number of methods exist for determining the decomposition in which the potential is defined; however, by far the most common is via the steady state probability distribution over the state space, where *U* ∝ −ln(*P*_*s*_(*X*)). Numerous studies have obtained landscapes based upon this approach, either using methods that directly obtain the steady state distribution [25, 28, 29], or use extensive simulations to estimate the distribution empirically [11, 30–32]. Alternative approaches include those based upon variational principles and the action in moving between any two points [12, 23, 28], empirical Lyapunov functions [33], approximations to the Lyapunov functions [34], or an orthogonal decomposition of the vector field [35]. Regardless of the method, the concept of dynamics composed of both a potential and vector field is commonplace, and the potential is indeed related to the idea of an epigenetic landscape.

In order to elucidate the distinction between gradient-based and rotational dynamics we first examine a simple bistable system expressed in SDE form. We define the system in terms of a pre-specified two-dimensional potential *U*(*X*; *θ*), chosen as,

$$\begin{array}{c} U\left(x_{1},x_{2}\right)=\lambda \left(x_{1}^{4}+x_{2}^{4}\right)-\alpha x_{1}^{2}+\beta x_{1}. \end{array}$$ (4)

**Fig 1:**
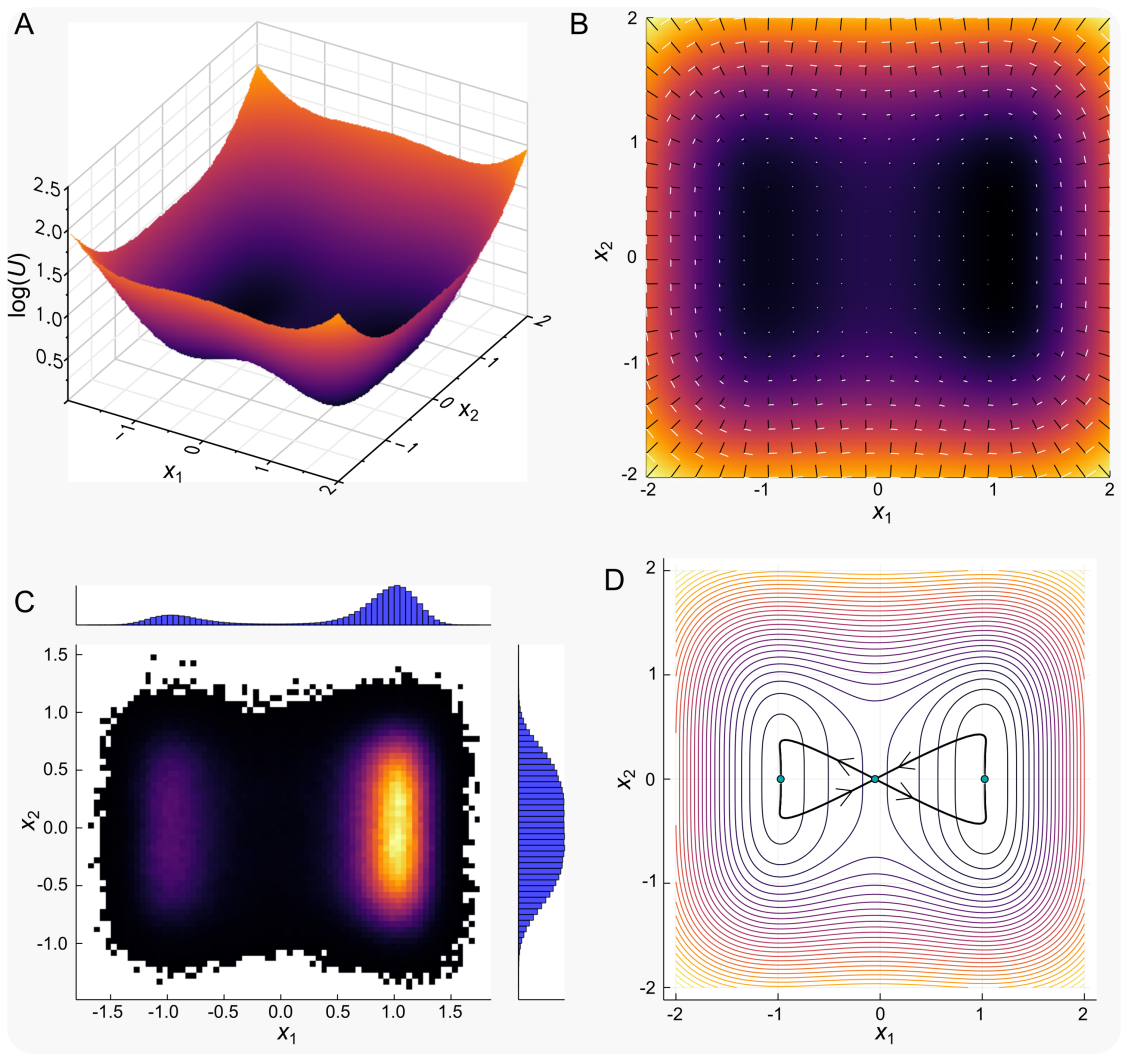
The two-dimensional SDE model. (A) surface plot of the potential field *U*; (B) the vector field decomposed into the gradient (black) and curl (white) components; (C) the two-dimensional histogram generated from a long-run simulation of the system; (D) The minimum action paths (MAP) between stationary points *X*_*i*_. On exit from an attractor the MAP follows a path $\dot{X}=\nabla U+f_{U}$, before following a “free fall” path back towards a stationary point.

This potential is displayed in Fig 1A and 1B and can be seen to consist of two minima at (*x*_1_, *x*_2_) ≈ (±1, 0) and one saddle point at (*x*_1_, *x*_2_) ≈ (0, 0). Given such a potential field, we may then define the vector field *f*(*X*; *θ*) as the sum of −∇*U* and a component *f*_*U*_ chosen here to be such that ∇*U* ⋅ *f*_*U*_ = 0. This choice of an orthogonal component is convenient for demonstration purposes but may not be possible for general vector fields [36]. Given the choice of *U* in (4), these two components are given as

$$-\nabla U=\left[\begin{array}{c} 2\alpha x_{1}-4\lambda x_{1}^{3}-\beta \\ -4\lambda x_{2}^{3} \end{array}\right],$$ (5)

$$f_{U}=C\left(X\right)\left[\begin{array}{c} 4\lambda x_{2}^{3}\\ 2\alpha x_{1}-4\lambda x_{1}^{3}-\beta \end{array}\right],$$ (6)

respectively, where *C*(*X*) can be any scalar state dependent function, chosen here to be a simple constant *c*. The vector field components shown in Fig 1B display the two components of *f*. The black vectors can be seen to point in the direction of decreasing *U* and represent the −∇*U* component. The white vectors represent the curl component of the vector field and are parallel to the contours of *U* due to the orthogonality. Together these components on average lead to spiralling paths leading towards the potential surface’s minima. With sufficient stochastic forcing the system may also make intermittent transitions between the two potential wells.

Fig 1C displays the probability distribution over the state space defined by the variables *x*_1_ and *x*_2_. The distribution is clearly strongly linked to the landscape displayed in Fig 1A and 1B, (it is in fact implied by it) and is seen to peak at (*x*_1_, *x*_2_) ≈ (±1, 0). By contrast the system spends relatively little time around the origin which is the third, unstable, fixed point of the system. It is worth noting that the distributions for the cases with and without a curl component are practically indistinguishable. This is typical for such systems and is the basis for identifying a landscape based upon the probability distribution alone [26]. However, a corollary of this is that only part of the dynamics are readily identifiable based upon static data [37]; in a biological context, knowing the transcriptomic states of cells tells us very little about the paths through transcription space taken during differentiation.

#### Static landscapes and the transition state

The simple model discussed above consists of a fixed potential *U*, parametrized by the vector *θ* = [λ, *α*, *β*]. This potential defines a *static* landscape since the potential itself remains constant with time. Given such a static landscape, we can define a transition state (TS) for the system based upon the most probable transition paths between the two stable fixed points. In the limit of small noise, the transition paths follow a particular route, that of *least action* [21]. These paths are known as the minimum action paths (MAP), and for a system in which the gradient and curl components are orthogonal, Freidlin and Wentzell further demonstrated that they follow a particular route: $\dot{X}=\nabla U+f_{U}$. These paths are displayed in Fig 1D.

The transition paths in both directions clearly pass through the unstable fixed point at *X* = (0, 0). This may therefore be identified as the transition state *X*_*t*_, and represents a location in state space that, with high probability, the system will pass through when transitioning between the two attractors of the system. In the context of stem-cell differentiation, such a state might represent a particular transcriptional configuration through which all cells transition when changing between a given two phenotypes. It is therefore important to consider which properties of the system determine how long is spent near this state, since this has implications for our ability to locate TSs, or map the path through gene expression space between cell fates from experimental single-cell data [5, 38, 39]

In a dynamical systems context, the linear stability of fixed point such as *X*_*t*_ can be determined from the Jacobian at this point. Here, we examine the eigenvalues of the Jacobian, which provide information regarding the local stability and typical trajectories following a small perturbation [40]. Eigenvalues with negative real part imply trajectories that decay towards the fixed point, while those with positive real part imply exponentially growing trajectories. In general, the real parts may be associated with the local landscape while the imaginary parts describe the locally orthogonal curl component.

**Fig 2:**
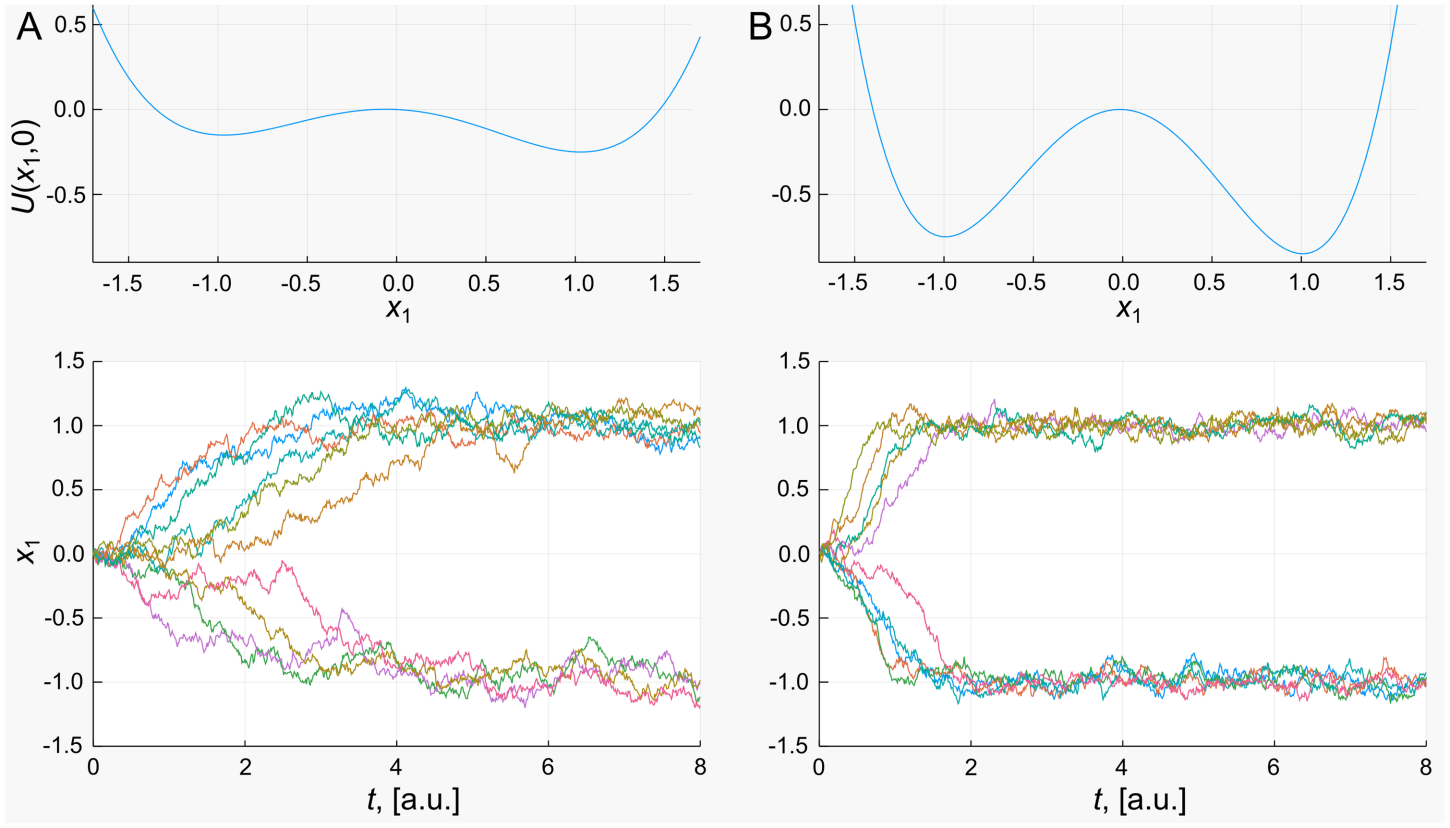
Influence of eigenvalues. Slice through the potential and example trajectories away from the transition state under different model parameters: (A) small positive eigenvalue (*α* = 0.4); (B) large positive eigenvalue (*α* = 1.6). For a larger positive eigenvalue the wells of the system are deeper and the curvature is greater, leading to more rapid movement away from the transition state.

For our example defined by Eq 5, the positive real eigenvalue may be shown to be determined by the parameter *α*. We therefore simulate the system with two different values of *α*, while keeping the ratio *α*/λ constant. Example trajectories from such simulations are displayed in Fig 2.

The trajectories shown in Fig 2A, while all starting from the same initial conditions display high variability due to the stochastic nature of the system. Paths diverge towards one of the two stable equilibria for which *x*_1_ ≈ ±1. The rate at which this divergence occurs varies considerably, although most seem to have undergone this divergence after eight time units. By contrast, the trajectories in Fig 2B diverge towards the stable states in much less time, although the relative variability is similar. This may be attributed to the larger positive eigenvalue which governs the time-scale of the process. This is in turn related to the shape of the landscape as also shown in Fig 2: a larger positive eigenvalue leads both to a higher barrier in the potential between the two stable states as well as steeper roll off. The time spent at the transition state must reflect the curvature of the landscape, which in turn is quantified by the eigenvalues of the Jacobian.

The results of Fig 2 demonstrate that once the transition state is reached, the time spent there depends upon the shape of the landscape. Another important consideration is the frequency with which the TS might be reached, and therefore transitions between attractors can occur. This frequency is dependent on both the shape of the landscape and the magnitude of the noise, quantified by *σ*, and for simple systems may be expressed directly in terms of these parameters [41]. As would be expected, transitions become less likely with increasing *α* as the wells of the landscape become deeper, while with increasing *σ* transitions become more likely as the noise can more readily perturb the system out of the basins of attraction.

#### Transitory landscapes: The competing definition for a transition state

While the system described above consists of a static landscape, a modified system might be formed by having parameters change over time. Here *β* alters the landscape, shifting which stable state is preferred. A landscape where parameters change may be referred to as *transitory*, as the shape of the potential is time-varying. In the context of epigenetic landscapes, the transitory interpretation gives an alternative viewpoint somewhat contrary to Waddington’s original, yet not without precedent in the literature [12, 42].

By varying *β* over a suitable range, the system changes from one in which there is a single stable equilibrium at positive *x*_1_ to the bistable system displayed in Fig 1, to one with a single stable equilibrium at negative *x*_1_.

**Fig 3:**
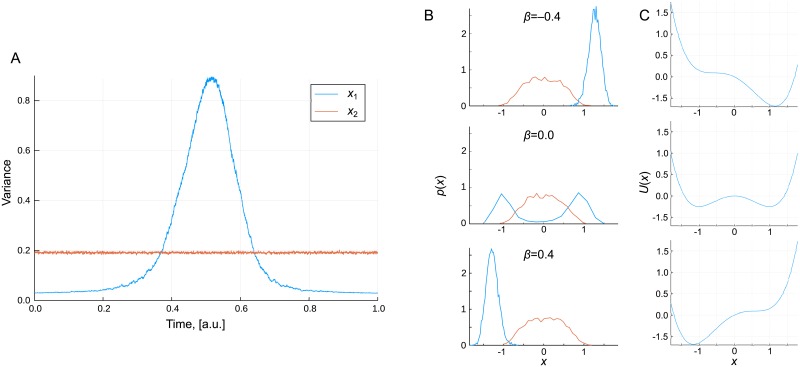
The transition state in a transitory landscape. (A) The variance of the two states across 5000 simulations in which *β* is linearly varied with time; (B) Probability distributions of the two states at three different values of *β*; (C) The potential *U* at each of the three values of *β*. The high variance “transition state” corresponds to intermediate values of *β* in which significant bistability generates large heterogeneity.

We demonstrate the concept of a transitory landscape by generating an ensemble of simulations in which *β* varies linearly over time from -0.5 to 0.5, (*β* = *t* − 0.5). Fig 3A shows the variance across this ensemble of simulations for the state variables *x*_1_, *x*_2_. While the variance of *x*_2_ remains constant, the variance of *x*_1_ can be seen to begin and end at very low values but transition through a time-period of high variance. This high variance is associated with the bistability of the system that is observed for small absolute values of the parameter *β*, as displayed in the probability distribution for which *β* = 0.0 in Fig 3B.

The time-period of high variance displayed in Fig 3A is analogous to the transition state concept posited by [5]. In the context of stem cell development, high variance across an ensemble of simulations is equivalent to high heterogeneity across a population of cells at the same developmental stage.

It is also worth noting at this point that the choice between a static or transitory landscape is really an issue of model complexity: as an alternative we can consider *β* to be an additional state rather than a model parameter. The system would therefore be three-dimensional with some additional dynamics governing how this third state *x*_3_ = *β* evolves with time. Both models will be able to capture the same behaviour but would differ in the way they described it: the landscape of the former being two-dimensional and time-varying while that of the latter being static and three-dimensional.

### The transition states and landscapes in a developmental model

**Fig 4:**
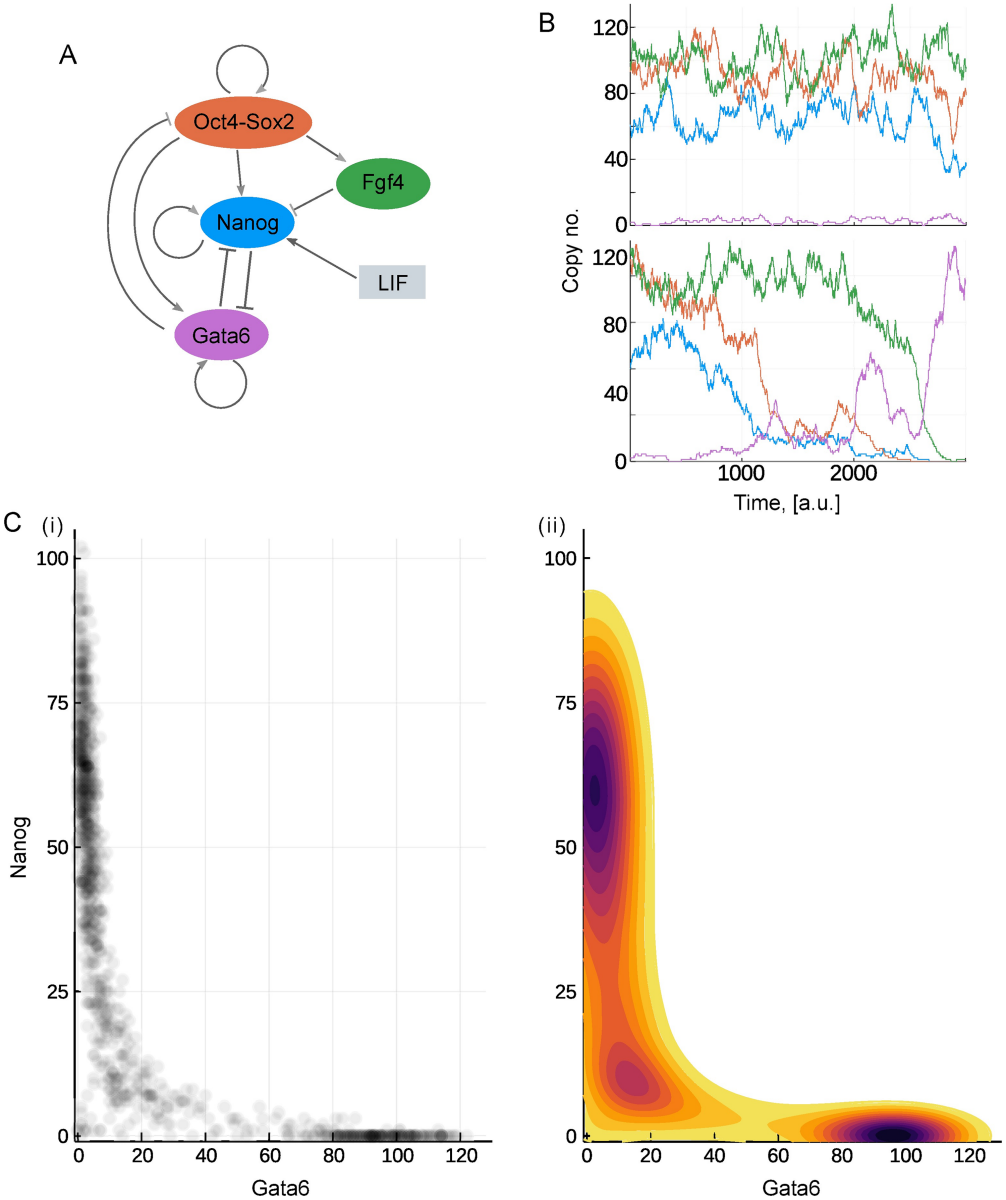
Potential landscape of the stem cell differentiation model. (A) Schematics of the stem cell differentiation model. (B) Simulated example of a cell that stays pluripotent (upper axes) and a cell undergoing differentiation (lower axes). The abundance of each species is indicated by a line coloured according to the filled ovals in (A). (C) Probability distribution and landscape of the system with *L* = 50. (i) Scatter plot of the whole population on the Gata6-Nanog plane. (ii) The landscape computed as the negative logarithm of the probability distribution.

With the insights derived from this illustrative model, we continue our analysis with a model that incorporates some of the dynamic relationships involved in stem cell differentiation [43]. The model focuses on four key players: Nanog (*N*), the complex Oct4-Sox2 (*O*), Fgf4 (*F*), and Gata6, (*G*) a typical differentiation marker. A schematic representation of their network is shown in Fig 4A; all arrows stand for non-linear interactions that account for implicitly modelled species and processes, like the formation of the Oct4-Sox2 complex, or Nanog dimerization. In addition, Leukaemia inhibitory factor (LIF), is also included in the model: the concentration of LIF (*L*) is treated as an external control, through which the environment of the cells is modified. Full details of the model are given in the methods section, while further analysis may be found in [44].

#### The probabilistic landscape and system behaviour

In this model, high expression of Nanog and Gata6 indicate stem cell and differentiated phenotypes respectively. Since there is mutual inhibition between these two molecules, a state where both are simultaneously high or low is unlikely. Fig 4B shows example simulations with *L* = 50 and intermediate initial expression levels, under which conditions many cells stay pluripotent (upper), but some stochastically shift into a differentiated state (lower). In general, simulations initiated with high values of Nanog, Fgf4 and Oct4-Sox2 will fluctuate around these high values, before ultimately switching to a configuration with high Gata6 and low expression levels of the other molecules. The time at which this switch occurs is highly variable, but the switch itself is seen to be irreversible, indicating that the differentiated state is in some sense more stable.

In order to gain further insight into the behaviour of the system we compute the probabilistic landscape for *L* = 50. This computation is achieved by performing a large number of simulations over a fixed time-period, from which the final expression levels are sampled (Fig 4C(i)). The landscape is then evaluated as minus the logarithm of this empirical distribution and is shown in Fig 4C(ii).

**Fig 5:**
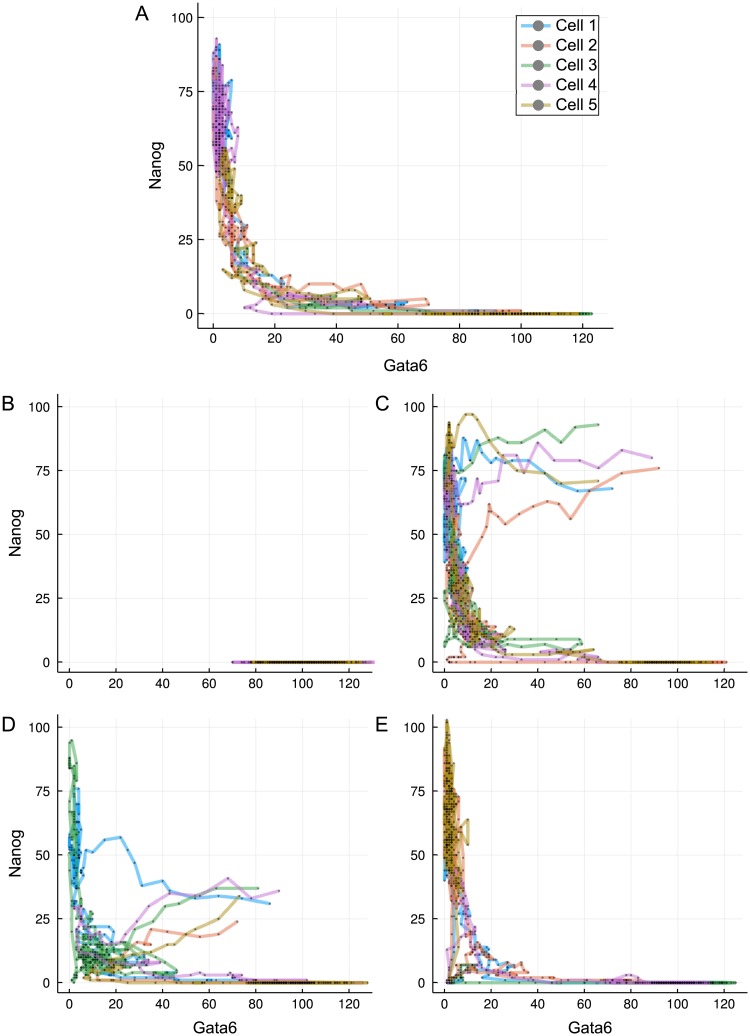
Transition paths during differentiation. (A) Trajectories of five randomly selected stem cells after reduction in LIF concentration (*L* = 0), plotted on the Gata6-Nanog phase space. (B) Cells started from the differentiated state and put into *L* = 200. (C-D) Cells started from the unexplored region of high Gata6 and high Nanog (C) or low Nanog (D) levels for *L* = 0. (E) Trajectories started from the transition state under *L* = 50. Different line colours denote different cells, as indicated in the legend.

The potential landscape displays a narrow valley which provides the most likely connection between the differentiated and pluripotent states. To compare the final potential landscape with paths taken by cells, we let cells differentiate *in silico*. First, we simulate a cell population in a high LIF medium, with initial values from the *N* ∈ [60, 100], *G* ∈ [0, 16] region and *L* = 200. We then change the external conditions, *L*: 200 → 0, and simulate the differentiation paths. As demonstrated by randomly selected trajectories in Fig 5A, the dynamical paths show good correspondence with the previously derived static landscape: cells traverse through the narrow valley between the stem cell and differentiated states.

Most of the time along typical cell paths is spent in either of the two valleys: cells linger in the stem cell valley for varying amount of time, but as their Nanog level decreases below a critical threshold, they quickly transit to the differentiated cell valley. Some trajectories do not exit the self-renewing state (such as cell 3 on panel A) over the simulated time-course, accounting for the less populated but still prevailing stem cell population in Fig 4C. We also test the system in a reversed differentiation scenario: cells already in the differentiated valley are simulated under high LIF conditions (*L* = 200) and tracked. The trajectories, shown in Fig 5B, confirm that once differentiation is achieved the cells cannot regain pluripotency, as consistent with an irreversible switch-like process.

As is clear from the derived landscape, the majority of the Gata6-Nanog phase space lies on a high-potential plateau that remains unexplored by the cells, displayed as the large white region of Fig 4C. Therefore, we have to artificially introduce conditions that allow the mapping of cell dynamics over this region of the phase space. We first uniformly sample cells from an uncovered volume of four-dimensional space, for example {*N*, *O*, *G*} ∈ [60, 100] and *F* ∈ [10, 50]. Next we follow the same experimental procedure as before and induce differentiation by setting *L* to 0. Surprisingly, trajectories from this region quickly converge into the Nanog-high valley and then follow the paths observed on a stem cell starting population, as displayed in Fig 5C.

Initial values sampled from another region of the high-potential plateau ({*O*, *F*, *G*} ∈ [60, 100]; *N* ∈ [20, 50]) result in trajectories that do not follow a direct route (Fig 5D). These cells first visit a state characterized by simultaneously low Gata6 and Nanog expression, and then follow the connecting valley to the differentiated cell state/phenotype. Such a state is reminiscent of the static transition state discussed above, while the phenomenon is consistent for a wide range of initial conditions, suggesting that any cell—unless it has already differentiated—has to go through the transition state. Cells that reside in the transition state, on the other hand, have the ability to progress towards either the stem cell or differentiated state, as seen in Fig 5E. This is in agreement with other models reporting a distinguished transition state along the way of cell specialization that has the potential for any cell fate [13, 45, 46].

#### Most probable paths in the static landscape

The indirect transition paths observed to be taken by the cells are indicative of a curl component in the dynamics. This finding does not contradict the landscape of Fig 4C since such components are not captured in the potential, but is illustrative of the ubiquity of such dynamics.

**Fig 6:**
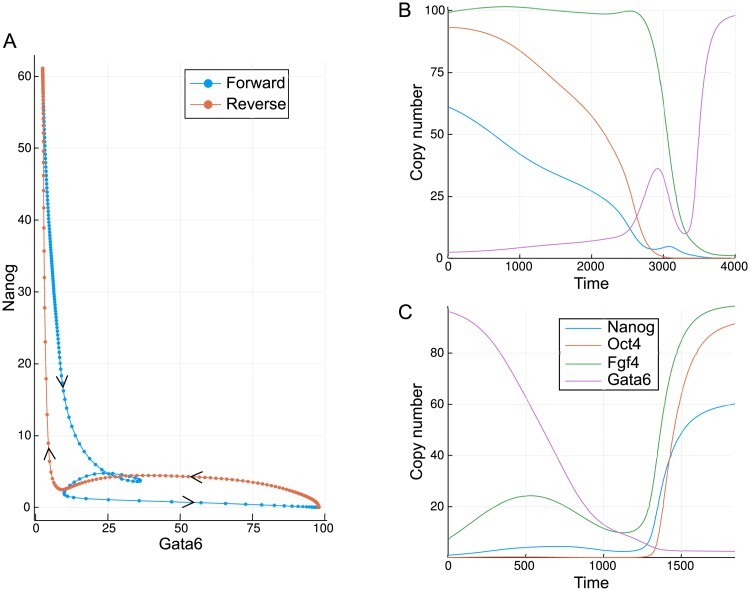
Minimum action paths for the developmental model. (A) A comparison of the paths plotted in the Gata6, Nanog plane; (B) Time series for each of the molecular species in the forward direction; (C) Time series for each of the molecular species in the reverse direction.

One implication arising from the presence of curl is that the optimal paths in the forward and reverse directions will not be identical, as was seen for the toy model in Fig 1D. For this system the optimal, or minimum action, path in the forward direction may in principle be inferred from simulations, since this path is also the most probable. However in practice the high variability between simulations makes the precise path difficult to observe. In the reverse direction, simulations may never yield a path, and the hypothetical MAP must instead be evaluated via an optimization approach, as detailed in [47]. The MAPs in each direction are displayed in Fig 6.

As is clear from Fig 6A, the paths in the forward and reverse directions are different, although both traverse the valley-like region of the Gata6-Nanog landscape shown in Fig 4D. While the paths are similar, it is important to note that in the four-dimensional space of this model, they never meet except at the start and end points. However both paths come close to each other near to the static transition state at low values of both Gata6 and Nanog. While the reverse path travels to and from the transition state with more-or-less direct trajectories, the forward path is seen to loop around in a convoluted manner. Such behaviour is indicative of curl dynamics and is consistent with the complex eigenvalues of the system, as will be discussed below. The differences between the forward and backward paths may be seen more clearly in the time-series. In the forward direction, the MAP begins with a slight transient increase in Fgf4 accompanying a gradual fall in Nanog and Oct4 (Fig 6B). Gata6 rises slowly at first before rising much more rapidly once Nanog is below a critical level. The mutual inhibition between Nanog and Gata6 leads to a similar effect in the reverse direction: Gata6 must fall rapidly, and only once it is below a fairly low level (≈ 10) does the abundance of the other species begin to rise (Fig 6C).

The key observation from the MAPs is that because of the curl component to the dynamics, the most probable paths and static transition states are different for each direction. Thus, if we wish to gain insight into the easiest routes for the reprogramming of cells back to pluripotency, observation of the typical forward paths will likely not be sufficient. Yet despite the differences, the paths may meet or come close together at a transition state such as a saddle point in the landscape [28], as was the case here.

### Transitory landscapes in a time-varying model

The behaviour illustrated above suggests the existence of a transition state in the static landscape, similar to the saddle point in the toy model discussed above. However, the transition state may also be defined for a transitory landscape, which in this case may be achieved by varying the level of LIF, quantified by the parameter *L*.

**Fig 7:**
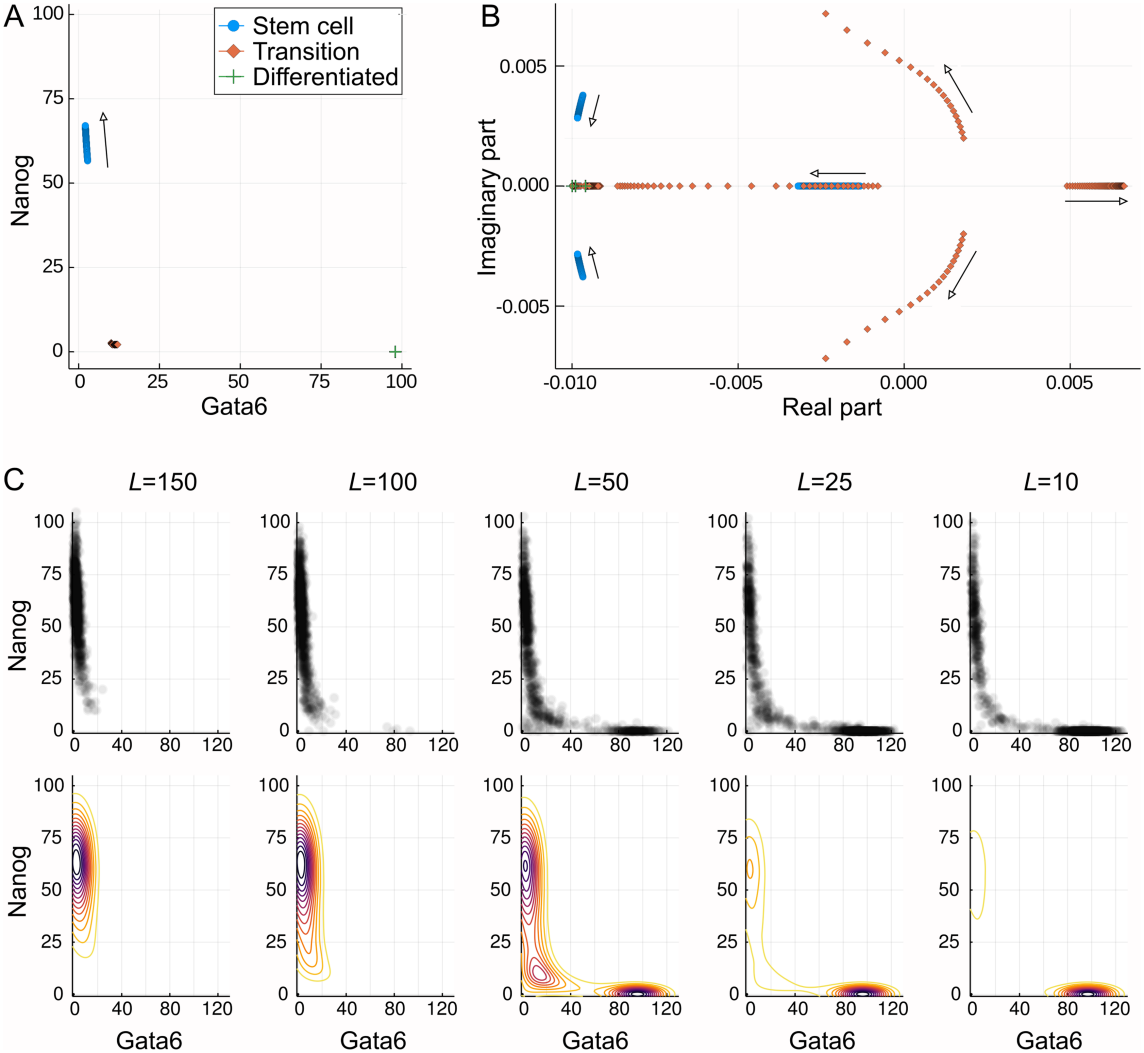
Varying behaviour of the system with LIF concentration, *L*. (A) Three of the fixed points of the model with increasing *L*. The differentiated and transition state remain unchanged while the stem cell state moves to higher Nanog values. (B) The eigenvalues of the Jacobian at each of the three fixed points as *L* increases. The stem cell state becomes locally more stable, as indicated by the increasingly negative eigenvalues. The transition state becomes increasingly unstable, thereby making transition to this state less probable. The differentiated state is unaffected, but is seen to be locally more stable than the stem cell state. Complex eigenvalues are associated with rotational “curl” dynamics. (C) Variation of the probabilistic landscape. Upper plots show a sample of the sampled points while lower plots show the landscapes. As *L* decreases the valley at the differentiated state becomes deeper while that at the stem cell state becomes shallower. At intermediate values the landscape is flatter around the transition state.

Before proceeding to simulations of a transitory system, it is first insightful to examine analytically how the fixed points (*X*_*i*_) of the system vary with *L*. This is achieved by finding the expression levels at which the rate equations are equal to zero. Three such fixed points are displayed in Fig 7A for varying *L*. The three identified fixed points may be associated with each of the stem-cell, transition and differentiated states of the system. Of these three states it is only the stem-cell state that is strongly influenced, moving to higher values of Nanog as *L* is increased.

In addition to the location of the fixed points, we may also examine the eigenvalues of the Jacobian at each of them. These eigenvalues, over a range of LIF concentrations, are displayed in Fig 7B as a scatter plot in the complex plane. Because the system is four-dimensional, each fixed point has four eigenvalues associated with it, although these may sometimes be very close together. The eigenvalues for the stem cell and differentiated state can be seen to reside entirely in the left half plane, a characteristic indicative of the linear stability of these states. In contrast, the transition state is seen to always have at least one eigenvalue with positive real part, and is therefore unstable. The stem-cell and differentiated states are therefore attractors of the system while the transition state is a saddle-point in the landscape.

With increasing *L*, the magnitude of the positive real eigenvalue of the transition state increases. This implies both that the system will leave this state more rapidly if it passes through it, but also that arriving at this state is less probable, consistent with the observation that high *L* inhibits (initiation of) differentiation. In terms of the underlying landscape, the increasing positive eigenvalue corresponds to an increasingly steep roll-off away from the saddle point, just as for the toy model displayed in Fig 2.

The eigenvalues of the stem-cell state also vary with LIF concentration, in this case transitioning to complex values with increasingly negative real part. This implies increased stability of this system and, together with the variation of the transition state eigenvalues, is consistent with the behaviour of the system under increasing *L*: increased stability of the stem cell state.

The eigenvalues of the differentiated state do not vary with *L*, but all the values are seen to be more negative than some of those of the transition and stem cell states. This is consistent with the greater stability of the differentiated state and the tendency for the system to remain there once it has arrived. Furthermore, these properties of the different states may be related to the shape of the probabilistic landscapes shown in Fig 7C. As *L* varies the landscape is seen to transition from one with a single valley around the stem-cell state to one with two valleys of varying depth. Further to this, for the case of *L* = 50 the differentiated state is surrounded by more tightly spaced contours than is the stem-cell state, and hence higher local curvature; this is consistent with the relative size of the eigenvalues.

**Fig 8:**
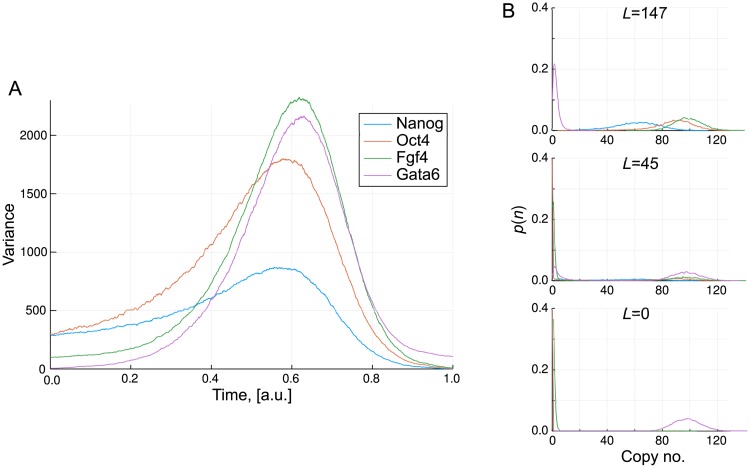
The transition state in a transitory landscape. (A) The variance of the transcription levels across 10,000 simulations in which *L* is linearly decreased with time as *L* = 150(1 − *t*); (B) Probability distributions of the transcription levels at three different values of *L*. The high variance “transition state” corresponds to intermediate values of *L* at which transitions from the stem cell to differentiated state begin to occur.

Given the observed variation of the system behaviour, we run simulations in which the parameter *L* is varied linearly over time. As for the toy model in section 1, we examine the variance across a large ensemble of simulations (10,000). This is displayed in Fig 8. The ensemble starts out with relatively low variance before displaying a period of high variance at intermediate times. In the context of real experimental data, such high variance would be observed as large heterogeneity across the sampled cells. This heterogeneity occurs because for this particular model, there is a distribution of times at which the stochastic switch is made from the stem-cell to differentiated state. Therefore even though all cells follow a similar path through gene expression space with very similar start and end points, there is high variability between cells over a particular period of time.

## Discussion

Models describing stem-cell differentiation are plentiful [48]. In this work we have examined one such model, that of [43], from a landscape perspective. Starting our analysis from an illustrative model we see that the landscape is strongly linked to the probability distribution over the state space, and gives an intuitive description of the dynamics. In the stem-cell differentiation model we are able to obtain a landscape that describes the typical system behavior. Moreover, the landscape may be linked to the dynamics in a quantitative manner via the fixed points *X*_*i*_ and the eigenvalues of the linearized dynamics at these locations: the fixed points occur at minima, maxima or saddle points in the landscape while the eigenvalues describe the local curvature.

While the potential landscape describes the tendency of the system to move towards particular stable states, it cannot fully capture all of the system’s dynamics. A non-gradient contribution to the dynamics is also generally present, known as the curl. A curl component is thought to be indicative of non-equilibrium systems, or those which are not closed to external influences [26]. This is because closed systems always have an available energy such as the Gibbs free energy which may be quantified and is directly related to the dynamics. For a developing stem cell, there is a constant exchange of both energy and nutrients via the diffusion of heat and mass through the membranes of the cell. Such a system is therefore clearly not closed in a thermodynamic sense, and non-equilibrium effects are bound to be present.

An alternative viewpoint on the presence of curl, is that a curl-free or purely gradient-based system requires complete symmetry in the interactions between all state variables [37]. In the context of gene regulatory networks, a necessary condition is that the influence of gene *i* on the expression of gene *j* is exactly the same as in the opposite direction. However even when this is the case, only in cases where these influences are of a particular functional form will the necessary symmetry be present. The precise requirement is that $\frac{\partial f_{i}}{\partial x_{j}}=\frac{\partial f_{j}}{\partial x_{i}}$, since both are equal to $\frac{\partial^{2}U}{\partial x_{i}\partial x_{j}}$. For systems such as a symmetric toggle switch in which there is mutual repression between two gene products, the standard Hill function form of the interaction does not lead to this symmetry. Therefore in general, with the exception cases in which the regulatory equations are specially chosen [49], we may expect a curl component to the dynamics of the system.

Although the potential landscape cannot describe all of the dynamics of cell differentiation, it can elucidate some pertinent features of the process. One such feature is the static transition state. Given a static landscape description for a developing cell, we may define transition states as the saddles in the landscape, corresponding to unstable fixed points of the associated dynamical system. The behavior of the system at these transition states is linked to the eigenvalues of the linearized dynamics, evaluated at these states. Such saddle points will always have eigenvalues with a positive real part, the magnitude of which is linked to the speed at which the transition states may be traversed. In the case that the transition speed is quick, the transition states may rarely be observed and measured trajectories will appear discontinuous, while if transitions are slow these states may correspond to the experimentally observed intermediate cell states.

The static transition states may also be inferred without recourse to the landscape, utilizing the concept of the minimum action path. Such paths have been evaluated for similar developmental models before [14], and are in general agreement with those found here. The MAPs describe the most probable routes through state space between any two fixed points. For purely gradient-based systems these routes simply follow that of steepest climb/descent and are therefore identical in both directions between any two points. For two-dimensional systems with an additional curl component such as the toy model examined above, the paths differ between the two directions but will meet at the saddle point between adjacent attractors. Such a saddle point may therefore be termed a static transition state. For higher dimensional systems such as the developmental model, the paths are again different but still come close together near to one of the unstable fixed points. The transcriptional region around this static transition state is therefore of significance for both differentiation and reprogramming.

An alternative to the static landscape description of the system is one of a transitory landscape, in which the potential changes under the influence of an external input or time-varying parameter. In this framework we may therefore consider an alternative definition for the transition state as those periods during development in which large heterogeneity is observed. Such heterogeneity may arise from two effects, firstly from the broad distribution of times for the transition from one fixed point to another, and secondly from a temporary flattening of the landscape under the influence of the changing conditions. In the models discussed here, both effects are present. The variation in switching times is typical of any stochastic system in which the escape from an attractor occurs due to random perturbations [41] and is therefore an inherent feature of many processes governed by a gene regulatory network. The temporary flattening of the landscape is dependent on the particular nature of the interactions between genes, but there is increasing experimental evidence for such a phenomenon [50]. The observation of cell-cell variability has become particularly apparent with the recent use of single cell experimental techniques. For example [51] observed high variability between cells undergoing transitions from one state to another, while [52] observed an increase in the variability between cells exiting pluripotency. Similar conclusions have also been drawn from the experimental work of [53] who suggested that cell fate transitions are linked to a critical change in the underlying landscape.

When forming any gene regulatory model, the choice between a static and transitory landscape may often be an issue of model complexity. For a gene regulatory network this choice is akin to that between modelling all of the relevant network interactions, or simply taking a subset of the network and treating the influence of other genes as (time-varying) parameters in the model. In the particular application of stem-cell development there is evidence that a relatively limited number of genes may be sufficient to describe typical behaviour, although external inputs are still required [54]. Such a system may therefore be described by a transitory landscape.

**Fig 9:**
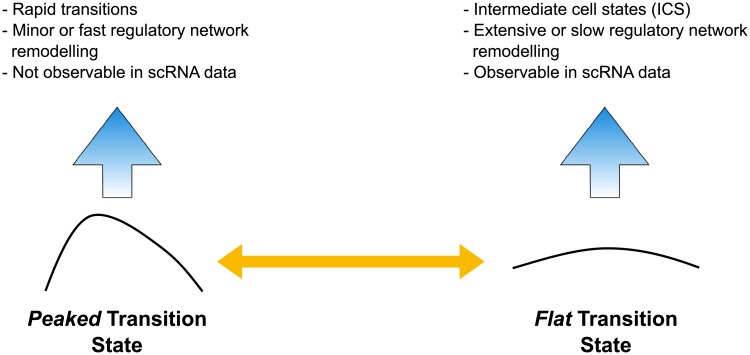
Relationship between transition state and observable data. The landscape curvature around the TS affects the time cells will spend in the vicinity of the TS, and reflects the molecular processes driving differentiation.

Elucidating the subtle relationship between transition states and intermediate cell states holds the key to linking mathematical models with data. TS are mathematically well defined unstable fixed points of a dynamical system; ICS are empirically determined accumulation points in gene expression space. Most likely they correspond to transition states with ‘flat’ local neighborhoods. Such landscape features will result in slower progress through the transition state/transitory region which may allow more substantial remodeling of the underlying transcriptional network [17, 39]. We can thus in principle use characteristics of the transition state or transitory region (see Fig 9) to demarcate two scenarios: (i) for a peaked TS we will see cells rapidly moving through the TS and being easily missed in single cell transcriptomic assays; (ii) for a flat TS we will observe ICS behavior [17] and are likely to capture some of the cells in the vicinity of the TS. This would clearly have implications for further analysis and network inference [55, 56], and will reflect the extent of network remodeling during differentiation, or the means by which this is achieved, e.g. 3D genome structure or epigenetic silencing/activation versus signaling events rapidly affecting transcription factor activity.

An important final consideration, irrespective of the transition state scenarios highlighted above, is that the presence of curl places limits on the identifiability of the dynamics. Most experimental data consists of purely static snapshots, from which instantaneous distributions can be obtained. Methods such as scRNA-seq only allow measurements of transcriptional profiles at single instances, and cannot provide the temporal variation of any individual cell. While this will enable measurement of the typical distribution of cell states, sometimes including the intermediate cell states, it will not readily allow measurement of certain characteristics of the cell paths, as discussed in [37]. The full identification of regulatory dynamics may therefore additionally require some measure of derivatives, as may be afforded by future experimental and analytical techniques [57]; this will still leave considerable challenges to identifying model structures [58].

## Methods

### Two-dimensional stochastic model

The two-dimensional toy model is chosen to exemplify some key properties of nonlinear stochastic dynamical systems. It is a two-dimensional extension of a well studied one-dimensional bistable system [41], known to exhibit a wide range of phenomena. The governing equations are,

$$\begin{array}{c} \text{d}X_{t}=[\begin{array}{c} 2\alpha x_{1}-4\lambda x_{1}^{3}-\beta +4c\lambda x_{2}^{3}\\ 2c\alpha x_{1}-4c\lambda x_{1}^{3}-c\beta -4\lambda x_{2}^{3} \end{array}]\;\text{d}t+[\begin{array}{cc} \sigma & 0\\ 0 & \sigma \end{array}]\;\text{d}W_{t}. \end{array}$$ (7)

Here d***W***_*t*_ refers to increments of a Wiener process, which can be interpreted as the time integral of Gaussian distributed white noise [59].

Unless otherwise stated, the parameter values used in the model were

$$\begin{array}{c} \left[\begin{array}{c} \alpha \\ \lambda \\ \beta \\ c\\ \sigma \end{array}]=[\begin{array}{c} 0.5\\ 0.25\\ -0.05\\ 0.5\\ 0.4 \end{array}\right] \end{array}$$

### The developmental model

The developmental model consists of four key players: Nanog (*N*) the complex Oct4-Sox2 (*O*), Fgf4 (*F*) and Gata6 (*G*). We additionally include the typical cell media LIF (*L*), which is a controllable parameter. Applying the quasi-equilibrium assumption, we treat all modifying interactions as factors in the same birth process, with their associated rates acting as a weighting on the net production rate. A first order degradation reaction parametrized by *k*_*d*_ is also included for each molecular species, leading to eight reactions with the following propensities:

$$\begin{array}{c} a_{1}=\frac{k_{0}O\left(k_{1}+k_{2}N^{2}+k_{0}O+k_{3}L\right)}{1+k_{0}O\left(k_{2}N^{2}+k_{0}O+k_{3}L+k_{4}F^{2}\right)+k_{5}OG^{2}} \end{array}$$ (8)

$$\begin{array}{c} a_{2}=\frac{k_{6}+k_{7}O}{1+k_{7}O+k_{8}G^{2}} \end{array}$$ (9)

$$\begin{array}{c} a_{1}=\frac{k_{9}+k_{10}O}{1+k_{10}O} \end{array}$$ (10)

$$\begin{array}{c} a_{1}=\frac{k_{11}+k_{12}G^{2}+k_{14}O}{1+k_{12}G^{2}+k_{13}N^{2}+k_{14}O} \end{array}$$ (11)

$$\begin{array}{c} a_{5}=k_{d}N \end{array}$$ (12)

$$\begin{array}{c} a_{6}=k_{d}O \end{array}$$ (13)

$$\begin{array}{c} a_{7}=k_{d}F \end{array}$$ (14)

$$\begin{array}{c} a_{8}=k_{d}G. \end{array}$$ (15)

The evolution of the system is then described by the combination of these eight reactions and a stoichiometry matrix *S*, that defines the integer changes to the copy number of each species. This is given as,

$$\begin{array}{c} S=[\begin{array}{cccccccc} 1 & 0 & 0 & 0 & -1 & 0 & 0 & 0\\ 0 & 1 & 0 & 0 & 0 & -1 & 0 & 0\\ 0 & 0 & 1 & 0 & 0 & 0 & -1 & 0\\ 0 & 0 & 0 & 1 & 0 & 0 & 0 & -1. \end{array}] \end{array}$$ (16)

The parameter values used in the model were

$$\begin{array}{c} {}[\begin{array}{c} k_{0}\\ k_{1}\\ k_{2}\\ k_{3}\\ k_{4}\\ k_{5}\\ k_{6}\\ k_{7}\\ k_{8}\\ k_{9}\\ k_{10}\\ k_{11}\\ k_{12}\\ k_{13}\\ k_{14}\\ k_{d} \end{array}]=[\begin{array}{c} 0.005\\ 0.01\\ 0.4\\ 1\\ 0.1\\ 0.00135\\ 0.01\\ 0.01\\ 1\\ 1\\ 0.01\\ 5\\ 1\\ 0.005\\ 1\\ 1 \end{array}]. \end{array}$$

### Numerical and analytical methods

Simulations of the developmental model were performed using the Gillespie algorithm [60], as is standard for chemical reaction systems. For the analysis of the fixed points, linearized dynamics and minimum action paths, the system was transformed into the approximate SDE format. Defining the vector *X* = [*N*, *O*, *F*, *G*]^⊤^, and the reaction vector ***a*** = [*a*_*i*_]^⊤^, the SDE approximation is given as [61]

$$\begin{array}{c} \text{d}X_{t}=Sa\;\text{d}t+S\text{diag}(\sqrt{a})\;\text{d}W_{t}. \end{array}$$ (17)

Here diag(***v***) stands for the $R^{N\times N}$ matrix with the elements of vector ***v*** in its diagonal and zeros elsewhere.

The deterministic fixed points of the system may be obtained by solving the equation,

$$\begin{array}{c} f(X)=Sa(X)=0. \end{array}$$ (18)

Due to the nonlinear nature of the equations, analytical solutions are difficult to obtain. Solutions were therefore found numerically using the DifferentialEquations.jl package in Julia [62]. Analysis of the Jacobians was performed symbolically using the SymPy.jl package.

The minimum action paths were computed via the optimization approach detailed in the supplementary information of [47]. The MAP are those paths through the state space that minimize the Freidlin-Wentzell action functional [21].

## References

[1] Waddington CH. The strategy of the genes: a discussion of some aspects of theoretical biology. London: Allen & Unwin; 1957.

[2] Filippi S, Barnes CP, Kirk PDW, Kudo T, Kunida K, McMahon SS, et al Robustness of MEK-ERK Dynamics and Origins of Cell-to-Cell Variability in MAPK Signaling. Cell Reports. 2016;15(11):2524–2535. 10.1016/j.celrep.2016.05.024 27264188 PMC4914773

[3] Hormoz S, Singer ZS, Linton JM, Antebi YE, Shraiman BI, Elowitz MB. Inferring Cell-State Transition Dynamics from Lineage Trees and Endpoint Single-Cell Measurements. Cell Systems. 2016;3(5):419–433.e8. 10.1016/j.cels.2016.10.015 27883889 PMC5142829

[4] Moris N, Martinez-Arias A. The Hidden Memory of Differentiating Cells. Cell Systems. 2017;5(3):163–164. 10.1016/j.cels.2017.09.009 28957650

[5] Moris N, Pina C, Arias AM. Transition states and cell fate decisions in epigenetic landscapes. Nature Reviews Genetics. 2016;(11):693–703. 10.1038/nrg.2016.98 27616569

[6] Huang S, Li F, Zhou JX, Qian H. Processes on the emergent landscapes of biochemical reaction networks and heterogeneous cell population dynamics: differentiation in living matters. Journal of the Royal Society Interface. 2017;14(130):20170097 10.1098/rsif.2017.0097 PMC5454298 28490602

[7] Pujadas E, Feinberg AP. Regulated noise in the epigenetic landscape of development and disease. Cell. 2012;148(6):1123–31. 10.1016/j.cell.2012.02.045 22424224 PMC3488344

[8] Feinberg AP, Koldobskiy MA, Gondor A. Epigenetic modulators, modifiers and mediators in cancer aetiology and progression. Nature Reviews: Genetics. 2016;17(5):284–299. 10.1038/nrg.2016.13 26972587 PMC4888057

[9] Teramoto Y, Takahashi DY, Holmes P, Ghazanfar AA. Vocal development in a Waddington landscape. eLife. 2017;6:e20782 10.7554/eLife.20782 28092262 PMC5310845

[10] Sasai M, Wolynes PG. Stochastic gene expression as a many-body problem. Proceedings of the National Academy of Sciences, USA. 2003;100(5):2374–9. 10.1073/pnas.2627987100 PMC151348 12606710

[11] Wang J, Xu L, Wang E, Huang S. The potential landscape of genetic circuits imposes the arrow of time in stem cell differentiation. Biophysical Journal. 2010;99(1):29–39. 10.1016/j.bpj.2010.03.058 20655830 PMC2895388

[12] Wang J, Zhang K, Xu L, Wang E. Quantifying the Waddington landscape and biological paths for development and differentiation. Proceedings of the National Academy of Sciences, USA. 2011;108(20):8257–62. 10.1073/pnas.1017017108 PMC3100956 21536909

[13] Li C, Wang J. Quantifying Waddington landscapes and paths of non-adiabatic cell fate decisions for differentiation, reprogramming and transdifferentiation. Journal of The Royal Society Interface. 2013;10(89):20130787 10.1098/rsif.2013.0787 PMC3808556 24132204

[14] Zhang B, Wolynes PG. Stem cell differentiation as a many-body problem. Proceedings of the National Academy of Sciences, USA. 2014;111(28):10185–90. 10.1073/pnas.1408561111 PMC4104876 24946805

[15] Li C, Wang J. Quantifying the Landscape for Development and Cancer from a Core Cancer Stem Cell Circuit. Cancer Research. 2015;75(13):2607–18. 10.1158/0008-5472.CAN-15-0079 25972342

[16] Yamanaka S. Elite and stochastic models for induced pluripotent stem cell generation. Nature. 2009;460(7251):49–52. 10.1038/nature08180 19571877

[17] MacLean AL, Hong T, Nie Q. Exploring intermediate cell states through the lens of single cells. Current Opinion in Systems Biology. 2018;9:32–41. 10.1016/j.coisb.2018.02.009 PMC6238957 30450444

[18] Hong T, Watanabe K, Ta CH, Villarreal-Ponce A, Nie Q, Dai X. An Ovol2-Zeb1 Mutual Inhibitory Circuit Governs Bidirectional and Multi-step Transition between Epithelial and Mesenchymal States. PLOS Computational Biology. 2015;11(11):e1004569 10.1371/journal.pcbi.1004569 26554584 PMC4640575

[19] Olsson A, Venkatasubramanian M, Chaudhri VK, Aronow BJ, Salomonis N, Singh H, et al Single-cell analysis of mixed-lineage states leading to a binary cell fate choice. Nature. 2016;537(7622):698–702. 10.1038/nature19348 27580035 PMC5161694

[20] Peine M, Rausch S, Helmstetter C, Fröhlich A, Hegazy AN, Kühl AA, et al Stable T-bet+GATA-3+ Th1/Th2 Hybrid Cells Arise In Vivo, Can Develop Directly from Naive Precursors, and Limit Immunopathologic Inflammation. PLOS Biology. 2013;11(8):e1001633 10.1371/journal.pbio.1001633 23976880 PMC3747991

[21] Freidlin MI, Wentzell AD, Szücs J. Random perturbations of dynamical systems. 3rd ed 260 Heidelberg [u.a]: Springer; 2012.

[22] Li C, Wang J. Quantifying Cell Fate Decisions for Differentiation and Reprogramming of a Human Stem Cell Network: Landscape and Biological Paths. PLOS Computational Biology. 2013;9(8):e1003165 10.1371/journal.pcbi.1003165 23935477 PMC3731225

[23] Wang J, Zhang K, Wang E. Kinetic paths, time scale, and underlying landscapes: A path integral framework to study global natures of nonequilibrium systems and networks. The Journal of Chemical Physics. 2010;133(12):125103 10.1063/1.3478547 20886967

[24] Heymann M, Vanden-Eijnden E. The geometric minimum action method: A least action principle on the space of curves. Communications on Pure and Applied Mathematics. 2008;61(8):1052–1117. 10.1002/cpa.20238

[25] Li C, Hong T, Nie Q. Quantifying the landscape and kinetic paths for epithelial–mesenchymal transition from a core circuit. Physical Chemistry Chemical Physics. 2016;18(27):17949–17956. 10.1039/c6cp03174a 27328302 PMC4948291

[26] Wang J. Landscape and flux theory of non-equilibrium dynamical systems with application to biology. Advances in Physics. 2015;64(1):1–137. 10.1080/00018732.2015.1037068

[27] Zhou P, Li T. Construction of the landscape for multi-stable systems: Potential landscape, quasi-potential, A-type integral and beyond. The Journal of Chemical Physics. 2016;144(9):094109 10.1063/1.4943096 26957159

[28] Lv C, Li X, Li F, Li T. Constructing the Energy Landscape for Genetic Switching System Driven by Intrinsic Noise. PLOS ONE. 2014;9(2):e88167 10.1371/journal.pone.0088167 24551081 PMC3923795

[29] Ge H, Qian H, Xie XS. Stochastic Phenotype Transition of a Single Cell in an Intermediate Region of Gene State Switching. Physical Review Letters. 2015;114(7). 10.1103/PhysRevLett.114.078101 25763973

[30] Wang J, Xu L, Wang E. Potential landscape and flux framework of nonequilibrium networks: Robustness, dissipation, and coherence of biochemical oscillations. Proceedings of the National Academy of Sciences, USA. 2008;105(34):12271–12276. 10.1073/pnas.0800579105 PMC2527901 18719111

[31] Li C, Wang E, Wang J. Potential Landscape and Probabilistic Flux of a Predator Prey Network. PLOS ONE. 2011;6(3):e17888 10.1371/journal.pone.0017888 21423576 PMC3058052

[32] Guo J, Lin F, Zhang X, Tanavde V, Zheng J. NetLand: quantitative modeling and visualization of Waddington’s epigenetic landscape using probabilistic potential. Bioinformatics. 2017; p. btx022 10.1093/bioinformatics/btx022 PMC5423452 28108450

[33] Bhattacharya S, Zhang Q, Andersen ME. A deterministic map of Waddington’s epigenetic landscape for cell fate specification. BMC Systems Biology. 2011;5(1):85 10.1186/1752-0509-5-85 21619617 PMC3213676

[34] Brackston RD, Wynn A, Stumpf MPH. Construction of quasipotentials for stochastic dynamical systems: An optimization approach. Physical Review E. 2018; 98(2):022136 10.1103/PhysRevE.98.022136 30253467

[35] Zhou JX, Aliyu MDS, Aurell E, Huang S. Quasi-potential landscape in complex multi-stable systems. Journal of the Royal Society, Interface. 2012;9(77):3539–3553. 10.1098/rsif.2012.0434 22933187 PMC3481575

[36] Brackston RD, Wynn A, Stumpf MPH. Construction of quasi-potentials for stochastic dynamical systems: An optimization approach. arXiv:1805.07273. 2018;. 10.1103/PhysRevE.98.022136 30253467

[37] Weinreb C, Wolock S, Tusi BK, Socolovsky M, Klein AM. Fundamental limits on dynamic inference from single-cell snapshots. Proceedings of the National Academy of Sciences, USA. 2018;115(10):E2467–E2476. 10.1073/pnas.1714723115 PMC5878004 29463712

[38] Moignard V, Woodhouse S, Haghverdi L, Lilly AJ, Tanaka Y, Wilkinson AC, et al Decoding the regulatory network of early blood development from single-cell gene expression measurements. Nature Biotechnology. 2015;33(3):269–276. 10.1038/nbt.3154 25664528 PMC4374163

[39] Stumpf PS, Smith RCG, Lenz M, Schuppert A, Müller FJ, Babtie AC, et al Stem Cell Differentiation as a Non-Markov Stochastic Process. Cell Systems. 2017;5(3):268–282.e7. 10.1016/j.cels.2017.08.009 28957659 PMC5624514

[40] Kwon C, Ao P, Thouless DJ. Structure of stochastic dynamics near fixed points. Proceedings of the National Academy of Sciences, USA. 2005;102(37):13029–13033. 10.1073/pnas.0506347102 PMC1201617 16141337

[41] Gammaitoni L, Hänggi P, Jung P, Marchesoni F. Stochastic resonance. Reviews of Modern Physics. 1998;70(1):223–287. 10.1103/RevModPhys.70.223

[42] Rabajante JF, Babierra AL. Branching and oscillations in the epigenetic landscape of cell-fate determination. Progress in Biophysics and Molecular Biology. 2015;117(2-3):240–249. 10.1016/j.pbiomolbio.2015.01.006 25641423

[43] Chickarmane V, Olariu V, Peterson C. Probing the role of stochasticity in a model of the embryonic stem cell—heterogeneous gene expression and reprogramming efficiency. BMC Systems Biology. 2012;6(98). 10.1186/1752-0509-6-98 22889237 PMC3468383

[44] Lakatos E. Stochastic analysis and control methods for molecular cell biology [PhD]. Imperial College London; 2017. Available from: http://hdl.handle.net/10044/1/53075.

[45] Martinez Arias A, Hayward P. Filtering transcriptional noise during development: concepts and mechanisms. Nature Reviews: Genetics. 2006;7(1):34–44. 10.1038/nrg1750 16369570

[46] Muñoz Descalzo S, Martinez Arias A. The structure of Wntch signalling and the resolution of transition states in development. Seminars in Cell & Developmental Biology. 2012;23(4):443–9. 10.1016/j.semcdb.2012.01.012 22326376

[47] Perez-Carrasco R, Guerrero P, Briscoe J, Page KM. Intrinsic Noise Profoundly Alters the Dynamics and Steady State of Morphogen-Controlled Bistable Genetic Switches. PLOS Computational Biology. 2016;12(10):e1005154 10.1371/journal.pcbi.1005154 27768683 PMC5074595

[48] Herberg M, Roeder I. Computational modelling of embryonic stem-cell fate control. Development. 2015;142(13):2250–2260. 10.1242/dev.116343 26130756

[49] Olariu V, Manesso E, Peterson C. A deterministic method for estimating free energy genetic network landscapes with applications to cell commitment and reprogramming paths. Royal Society Open Science. 2017;4(6):160765 10.1098/rsos.160765 28680655 PMC5493897

[50] Martinez Arias A, Brickman JM. Gene expression heterogeneities in embryonic stem cell populations: origin and function. Current Opinion in Cell Biology. 2011;23(6):650–656. 10.1016/j.ceb.2011.09.007 21982544

[51] Richard A, Boullu L, Herbach U, Bonnafoux A, Morin V, Vallin E, et al Single-Cell-Based Analysis Highlights a Surge in Cell-to-Cell Molecular Variability Preceding Irreversible Commitment in a Differentiation Process. PLOS Biology. 2016;14(12):e1002585 10.1371/journal.pbio.1002585 28027290 PMC5191835

[52] Semrau S, Goldmann JE, Soumillon M, Mikkelsen TS, Jaenisch R, van Oudenaarden A. Dynamics of lineage commitment revealed by single-cell transcriptomics of differentiating embryonic stem cells. Nature Communications. 2017;8(1). 10.1038/s41467-017-01076-4 29061959 PMC5653659

[53] Mojtahedi M, Skupin A, Zhou J, Castaño IG, Leong-Quong RYY, Chang H, et al Cell Fate Decision as High-Dimensional Critical State Transition. PLOS Biology. 2016;14(12):e2000640 10.1371/journal.pbio.2000640 28027308 PMC5189937

[54] Dunn SJ, Martello G, Yordanov B, Emmott S, Smith AG. Defining an essential transcription factor program for naive pluripotency. Science. 2014;344(6188):1156–1160. 10.1126/science.1248882 24904165 PMC4257066

[55] Babtie AC, Chan TE, Stumpf MPH. Learning regulatory models for cell development from single cell transcriptomic data. Current Opinion in Systems Biology. 2017;5:72–81. 10.1016/j.coisb.2017.07.013

[56] Chan TE, Stumpf MPH, Babtie AC. Gene Regulatory Network Inference from Single-Cell Data Using Multivariate Information Measures. Cell Systems. 2017;5(3):251–267.e3. 10.1016/j.cels.2017.08.014 28957658 PMC5624513

[57] La Manno G, Soldatov R, Hochgerner H, Zeisel A, Petukhov V, Kastriti M, et al. RNA velocity in single cells. bioRxiv. 2017 10.1038/s41586-018-0414-6 PMC6130801 30089906

[58] Babtie AC, Kirk P, Stumpf MPH. Topological sensitivity analysis for systems biology. Proceedings of the National Academy of Sciences, USA. 2014;111(52):18507–18512. 10.1073/pnas.1414026112 PMC4284538 25512544

[59] Gardiner CW. Stochastic methods: a handbook for the natural and social sciences 4th ed Springer series in synergetics. Berlin: Springer; 2009.

[60] Gillespie DT. A general method for numerically simulating the stochastic time evolution of coupled chemical reactions. Journal of Computational Physics. 1976;22(4):403–434. 10.1016/0021-9991(76)90041-3

[61] Gillespie DT. The chemical Langevin equation. The Journal of Chemical Physics. 2000;113(1):297–306. 10.1063/1.481811

[62] Rackauckas C, Nie Q. DifferentialEquations.jl—A Performant and Feature-Rich Ecosystem for Solving Differential Equations in Julia. Journal of Open Research Software. 2017;5 10.5334/jors.151

